# Household Air Pollution from Coal and Biomass Fuels in China: Measurements, Health Impacts, and Interventions

**DOI:** 10.1289/ehp.9479

**Published:** 2007-02-27

**Authors:** Junfeng (Jim) Zhang, Kirk R. Smith

**Affiliations:** 1 School of Public Health, University of Medicine and Dentistry of New Jersey, Piscataway, New Jersey, USA; 2 School of Public Health, University of California, Berkeley, California, USA

**Keywords:** burden of disease, cancer, household fuels, improved stoves, indoor air pollution, poisonous coals, respiratory disease

## Abstract

**Objective:**

Nearly all China’s rural residents and a shrinking fraction of urban residents use solid fuels (biomass and coal) for household cooking and/or heating. Consequently, global meta-analyses of epidemiologic studies indicate that indoor air pollution from solid fuel use in China is responsible for approximately 420,000 premature deaths annually, more than the approximately 300,000 attributed to urban outdoor air pollution in the country. Our objective in this review was to help elucidate the extent of this indoor air pollution health hazard.

**Data sources:**

We reviewed approximately 200 publications in both Chinese- and English-language journals that reported health effects, exposure characteristics, and fuel/stove intervention options.

**Conclusions:**

Observed health effects include respiratory illnesses, lung cancer, chronic obstructive pulmonary disease, weakening of the immune system, and reduction in lung function. Arsenic poisoning and fluorosis resulting from the use of “poisonous” coal have been observed in certain regions of China. Although attempts have been made in a few studies to identify specific coal smoke constituents responsible for specific adverse health effects, the majority of indoor air measurements include those of only particulate matter, carbon monoxide, sulfur dioxide, and/or nitrogen dioxide. These measurements indicate that pollution levels in households using solid fuel generally exceed China’s indoor air quality standards. Intervention technologies ranging from simply adding a chimney to the more complex modernized bioenergy program are available, but they can be viable only with coordinated support from the government and the commercial sector.

Although some areas of China are becoming more urban, more than 60% of the population is still rural, most of which still uses biomass (mainly wood and crop residues) and coal fuels that produce substantial pollution in simple stoves. In 2003 approximately 80% of the energy consumed by rural households was in the form of biomass and almost 10% as coal. Furthermore, although most Chinese cities have plans to eliminate coal for households, many urban communities continue to rely on coal. The combustion of biomass and coal (collectively called “solid fuels”) is the dominant source of indoor air pollution (IAP) in the country and contributes significantly to the total burden of ill health.

In the most recent global analysis of the health effects of major risk factors, the World Health Organization (WHO) estimated that solid fuels used in Chinese households cause approximately 420,000 premature deaths annually; this is 40% more than the approximately 300,000 premature deaths attributed to outdoor air pollution in Chinese cities with populations of more than 100,000 (Cohen et al. 2004; Smith et al. 2004). Household use of solid fuels is thus estimated to be the largest single environmental risk factor and ranks sixth among all risk factors examined for ill-health (Figure 1; Smith et al. 2005). These risk estimates, however, were based primarily on studies in other countries because, as discussed in this review, few epidemiologic studies have been conducted in China on biomass smoke compared with those conducted on coal smoke.

In the 1980s China conducted more IAP measurements focused on household combustion than all other developing countries combined. Indeed, by the early 1990s, the results of more than 100 published studies were combined into a WHO database (Sinton et al. 1996). In contrast, the Chinese environmental health community conducted few IAP measurements in the 1990s (Saksena et al. 2003), but epidemiologic studies of coal smoke, mainly on cancer end points, continued and have resulted in a large body of evidence. In particular, over 25 years a wide-ranging set of studies has been conducted on coal smoke exposures, toxicology, and health effects in one rural area—Xuanwei in Yunnan Province—with severe impacts.

In this review we address the following questions in order to put Chinese household IAP from solid fuel use into perspective, nationally and internationally, and highlight research gaps: *a*) What toxic constituents have been found in the emissions of solid fuel combustion? *b*) What are the reported human exposure characteristics? *c*) What health effects have been documented? and *d*) What technologies exist or are possible for reducing this IAP exposure?

## Methods

We conducted computer searches covering literature from 1980–2006 of the following bibliographic databases in English: PubMed (2007), Web of Science (2007), and Environmental Sciences and Pollution Management Index (2007); and in Chinese: China National Knowledge Infrastructure (1999) that contains 7,486 Chinese-language journals from 1979 to the present. We used the following key words individually or in combination: indoor air pollution, coal, biomass, and China. We also contacted several authors to obtain publications not readily available in the bibliographic databases. No attempt was made to search databases in other languages. Although no systematic screening procedures were possible, our review excludes much of the early literature because of uncertainties created by methodologic and reporting problems (Sinton et al. 1996; Smith and Liu 1994)

## Smoke Constituents

Solid fuels are difficult to burn in simple combustion devices such as household cooking and heating stoves without substantial emissions of pollutants, principally because of the difficulty of completely premixing the fuel and air during burning, which is done easily with liquid and gaseous fuels (Smith et al. 2000). Consequently, a substantial fraction of the fuel carbon is converted to products of incomplete combustion (PICs), namely, compounds other than the ultimate product of carbon dioxide that result from complete combustion. For example, household coal and biomass cookstoves in China divert more than 10% and up to 38% of their fuel carbon into PICs (Zhang et al. 2000). This is typical for simple stoves in households of developing countries (Smith et al. 2000).

PICs released from solid fuel combustion are a complex mixture of particulate and gaseous species. Some of the PICs are commonly regulated air pollutants such as carbon monoxide (CO), nitrogen dioxide (NO_2_), and particulate matter (PM). In studies characterizing PIC emissions from 28 fuel/stove combinations commonly found in China in the early 1990s, more than 60 hydrocarbons and 17 aldehydes and ketones were measured in larger quantities in the flue gases of solid fuel–burning stoves than in the flue gases of stoves burning liquid or gaseous fuel (Tsai et al. 2003; Zhang and Smith 1999). Identified gas-phase pollutants include compounds that are carcinogenic (benzene, formaldehyde), probably carcinogenic (1,3-butadiene), and possibly carcinogenic (styrene) to humans (Zhang and Smith 1996, 1999).

It is well known that polycyclic aromatic hydrocarbons (PAHs) are formed during incomplete combustion of all carbon-based fuels, including wood and coal. Lower molecular-weight PAHs (with two to four aromatic rings) are present predominantly in the gas phase, whereas higher molecular-weight PAHs are present predominantly in the particle phase. Because carcinogenic PAHs, especially benzo[*a*]pyrene (B[*a*]P), a 5-ring PAH of high cancer potency, are predominantly present in the particle phase, particles emitted from household coal combustion have been subjected to compositional analysis of PAHs and PAH derivatives. These analyses provide much needed information on the carcinogenicity and mutagenicity of coal smoke. For example, carcinogenic PAHs, methylated PAHs, and nitrogen-containing heterocyclic aromatic compounds were found in large abundance in the particles emitted from bituminous (smoky) coal combustion. Because carcinogenic PAHs, especially (B[*a*]P), a 5-ring PAH of high cancer potency, are predominantly present in the particle phase, particles emitted from household coal combustion have been subjected to compositional analysis of PAHs and PAH derivatives. These analyses provide much needed information on the carcinogenicity and mutagenicity of coal smoke. For example, carcinogenic PAHs, methylated PAHs, and nitrogen-containing heterocyclic aromatic compounds were found in large abundance in the particles emitted from bituminous (smoky) coal combustion, as is typically found in numerous households in Xuanwei (Chuang et al. 1992b; Granville et al. 2003; Keohavong et al. 2003; Mumford et al. 1987). These PAHs and PAH derivatives found in the coal smoke exhibited strong mutagenicity, and the subfractions containing alkylated three- and four-ring PAHs were found to contribute to most of the mutagenicity in the PAH fraction of coal combustion particles (Chuang et al. 1992a). In the aromatic fraction, coal combustion particles appear to contain higher concentrations and more species of methylated PAHs than wood combustion particles (Chuang et al. 1992b).

Unlike biomass, many coals contain intrinsic contaminants such as sulfur, arsenic, silica, fluorine, lead, mercury. During combustion these contaminants are not destroyed but are released into the air in their original or oxidized form. In households that use sulfur-rich coals, for example, sulfur dioxide (SO_2_) pollution affects not only indoor air quality (IAQ) but also outdoor air quality at a local or regional scale. Because coal burns at a substantially higher temperature than biomass, higher emissions of NO_2_ were measured for coal combustion than for biomass combustion (Zhang et al. 2000). Some carcinogenic substances in coal were released into the air during the combustion of lignites used in Shenyang city of northern China and smoky coals used in Xuanwei County. It has been reported that lignites from a local Shenyang coal field had the highest concentrations of nickel and chromium in the world (Ren et al. 1999, 2004). Microfibrous quartz has been found in some smoky coals and the resulting coal smoke in Xuanwei (Tian 2005). Particles emitted from burning coals contaminated with fluorine and arsenic in Guizhou Province and other areas contain high levels of these elements (Gu et al. 1990; Shraim et al. 2003; Yan 1990).

Although PM generated from the fuel combustion itself is fine and ultrafine in size, the smoke may contain larger particles resulting from suspension of ash and solid fuel debris. Because particle size determines how deep the particles can travel within and beyond the respiratory tract, determining size distribution is important in assessing health impacts. For this reason, in most recent studies there has been a switch to measuring inhalable particles [PM with an aerodynamic diameter ≤10 μm (PM_10_)] or respirable particles [PM with an aerodynamic diameter ≤2.5 μm (PM_2.5_)]. Although we could find no published studies on ultrafine particles [PM with an aerodynamic diameter ≤ 0.1 μm], it is expected that indoor levels of ultrafine particles would be high.

## Exposure Characteristics

Few studies have been conducted in China to measure personal exposures to solid fuel combustion products; thus measurements reported are typically of household indoor area concentrations. Recently, China pioneered IAQ standards [State Environmental Protection Agency of China (SEPA) 2007; Table 1] that address emissions in households. Although these standards can be used for comparison with measurements throughout the country, the impetus behind their creation was the growing concern in urban areas about what might be termed “modern” IAP, for example, emissions from furniture and building materials (Wang Z et al. 2004).

In most published studies of IAP health effects, exposure was assessed using crude proxies such as whether the households were using solid fuels. This has proved useful, but better quantifying exposure will be necessary for establishing exposure–response relationships and for more precisely quantifying health risks. In estimating the burden of disease from the solid fuel use in China where the household energy picture is quite complicated (with mixed use of different stove/fuels), one major uncertainty is the lack of accurate exposure data (Smith et al. 2004). To obtain population-based exposure data requires a well-planned integration of both indoor air concentration data and time–activity data specifically relevant to solid fuel use. Unfortunately, large-scale systematic and probability-weighted sampling of household IAP from solid fuel combustion has not been conducted to date in any developing countries, including China. Nevertheless, in approximately 120 studies, indoor concentrations of one or more pollutants were measured in one or more locations (e.g., kitchen, bedroom, living room, outdoors) within a household. Impressively, these studies covered rural and/or urban households of 29 provinces and municipalities of Beijing, Shanghai, Tianjin, and Chongqing (Supplementary Material; http://www.ehponline.org/docs/2007/9479/suppl.pdf). However, no standardized protocols were used, making cross-comparisons of the results from different studies difficult and possibly misleading.

Indoor concentrations depend on indoor emission rate of pollutant, air exchange rate, and room volume. Indoor emission rate can be largely reduced if there is a well-functioning flue to vent smoke outdoors, but flues are absent or poorly maintained in many Chinese households using solid fuels for cooking. In open fire Xuanwei households, mean indoor concentrations of PM_10_ were 24.4 mg/m^3^ during burning of smoky coal, 22.3 mg/m^3^ during burning of wood, and 1.8 mg/m^3^ during burning of anthracite (smokeless) coal (Mumford et al. 1987). Today in China tens of millions of people are still being exposed to the smoke emitted from open fire pits. Portable unvented coal stoves are quite common throughout much of the country for long-term cooking tasks such as keeping water hot for tea. Edwards et al. (2007) found that the use of such stoves indoors for 24 hr produces several hundred micrograms per cubic meter of fine particles. Reported concentrations of CO and SO_2_ also often exceed China’s IAQ standards, with the highest concentrations of CO and SO_2_ being 560 and 23 mg/m^3^, respectively (Sinton et al. 1996). A study comparing households using coal stoves and those using (liquified petroleum gas (LPG) stoves found 24-hr NO_2_ indoor concentrations significantly higher in the coal households (Zhang J et al. 1996). This is not surprising, as coal burning typically takes much longer than LPG or other gas burning for cooking.

House structure or room layout can significantly affect spatial and temporal distributions of pollutant concentrations within a household. For example, concentration differences between kitchens and living and sleeping rooms are generally greater in households with separate cooking and living and sleeping areas than in those without these areas, in the absence of heating source (Jin et al. 2005; Qin et al. 1991). In our review of studies in which air pollutants were measured in different rooms within the same households, we consistently found that concentrations of PM_10_ [or total suspended particles (TSPs)] and SO_2_ are highest in the kitchens of households using coal. This “kitchen effect” is, however, less profound and less consistent for NO_2_ and CO, perhaps because of the confounding from coal heating and tobacco smoking. Presumably, coal combustion for heating occurs at more steady burning conditions than for cooking. Peak emissions of particles occur during unsteady combustion stages such as at the beginning and at the end of the fire. These types of heterogeneous spatial and temporal patterns of pollutant concentration have important exposure implications for individual household members. A recent winter study in Jilin Province shows that households burning solid fuel had mean 24-hr levels of PM and CO that correlated with but were about 6 times lower than peak (1-hr) levels and that indoor levels were dominated by heating not cooking sources (Fischer and Koshland 2007). Having separate kitchens lowers pollutant concentrations in living/sleeping areas, thereby reducing general household exposure. As women typically cook in rural households, they might be expected to receive higher peak and cumulative exposures, although an exposure modeling assessment for Shanxi Province indicates that the difference between men and women may be minimal (Mestl et al. 2006).

An analysis of indoor–outdoor differences indicated that even when coal smoke is vented outdoors, indoor levels can be high in communities where large numbers of households use solid fuels because the “neighborhood” pollution created by local household emissions leads to significant re-entry of pollution back into the households (Smith et al. 1994). This is particularly important on cold winter days with poor atmospheric dispersion. (The most infamous air pollution episode in history, the London Smog of 1952, was largely due to this phenomenon.) Thus, a household using a clean fuel such as gas can still experience high indoor levels if located in a neighborhood with many solid fuel stoves.

Three recent projects have moved in the direction of using more standard methods and representative sampling techniques in a few rural areas and are summarized below.

### National Improved Stove Program (NISP) review

As part of a random 3,500-household survey, a random subsample of 396 rural households in provinces of Shaanxi, Hubei, and Zhejiang were monitored over a 24-hr period for PM_4_ (PM with aerodynamic diameter ≤4 μm) in kitchens and living rooms over (Edwards et al., in press). Among these 396 households, 159 were measured in both summer and winter. For nearly all household stove or fuel groupings, PM_4_ levels were higher than China’s IAQ standard for PM_10_ (SEPA 2007), even when the difference in particle size specified was ignored. Some of these higher levels were because of neighborhood pollution effects. Other higher levels were the result of the large variation in the complex fuel and stove situation in rural China where multiple types are used depending on season, room of the house, and purpose (cooking-water heating, space heating, heating beds). Many households change fuels according to daily and seasonal factors, resulting in different seasonal concentrations in living rooms, bedrooms, and kitchens. Indeed, 34 fuel–stove combinations were being used in some villages in the summer and 28 in the winter. The average household used 2.6 types of fuels. This complexity makes detailed assessment of the effects of different fuels, stoves, seasons, and household layout difficult without either large sample sizes in representative studies or more nonrepresentative studies in controlled settings. It illustrates that even though the “energy ladder” concept, in which households generally move from lower to higher quality household fuels with development (income), is well established at the macro level, the situation in individual households can be complex during the transition in a middle-income country like China (Sinton et al. 2004).

### World Bank Project

As part of a study funded by the World Bank, PM_4_, CO, and SO_2_ were monitored in 457 household-days in four poor provinces (Jin et al. 2005). The two provinces where biomass was the primary fuel had higher concentrations of PM_4_ and CO than the two primarily coal-burning provinces. Among the two coal-burning provinces, Guizhou had lower concentrations of CO and SO_2_ than Shaaxi. In addition to differences in local fuel quality and house ventilation conditions, heterogeneity in space heating contributes largely to the observed geographic differences in pollutant concentration within and between provinces. In the three northern provinces, indoor heating affected the level and spatial distributions of pollution inside homes, possibly to more of an extent than cooking. In an examination of temporal patterns of indoor pollution, day-today variability of concentrations within individual households, although substantial, were smaller than concentration variations across households. This temporal feature indicates that applying a crude proxy for exposure (e.g., whether or not using a biomass stove) would overlook large house-to-house differences in pollutant concentrations and exposures. It should be noted that although these studies employed a systematic measurement approach for all the measured households, the household selection procedure does not appear to be population-based or random; hence, the reported pollutant concentrations may not be generalizable to the general households in the four provinces. However, the spatial and temporal patterns observed in the study are expected to be typical for similar houses in the same regions (He et al. 2005; Jin et al. 2005).

### Sino-Dutch Project

The Sino–Dutch Project was a study of approximately 140 household kitchens in five counties of three provinces (Dong et al. 2006). The kitchens were monitored for 24 hr before and after introduction of a suite of improvements (improved biomass stoves, biogas) for PM_2.5_, CO, SO_2_, and ammonia. Measurements were performed a year apart to control for season and allowed for the improvements to “settle in.” Significant reductions occurred in all pollutants, with PM_2.5_ levels over a 24-hr period averaging about 120 μg/m^3^ after a year (Dong et al. 2006). The levels found in other studies indicated a reduction of only about 40% from preintervention values (Dong et al. 2006). Most households still did not meet the Chinese IAQ standard for PM_10_ (Dong et al. 2006).

A unique feature of coal smoke exposure in certain parts of China is the combination of inhalation exposure and ingestion exposure. Ingestion exposure occurs when foods are consumed that have been dried over coal or biomass fires. For example, in many “poisonous” coal endemic areas, drying corns and chili peppers over open fire pits is a common practice (He et al. 2005). During the drying process, the foods absorb the smoke and become enriched with toxic elements (e.g., fluoride and arsenic). The ingestion of contaminated foods is thought to be the predominant exposure route causing endemic arsenism and fluorosis in China (Chen et al. 1993; Finkelman et al. 1999; Jin et al. 2003). This potentially important exposure route, however, has not been examined for other toxic components (e.g., PAHs, nickel compounds) of coal or biomass smoke.

## Health Effects

Among the more than 100 papers reporting health effects of solid fuel combustion in Chinese households, most all have focused on coal use both in urban and rural populations. In contrast, the studies conducted in other developing countries have focused on households using biomass (Bruce et al. 2000; Smith et al. 2004). The large research effort on household coal smoke in China reflects the unique status of China as a “Coal Kingdom” where the use of household coal is spread widely throughout the country, with some of the coals containing toxic contaminants with unique health consequences. Household biomass use, however, is more prevalent and, as in the rest of the developing world, undoubtedly affects health as well, even though to date few studies of health effects have been conducted in China alone.

### Lung cancer

Associations between lung cancer and coal smoke exposure have been found in numerous epidemiologic studies conducted in China. Among these studies include the decades-long investigations in Xuanwei, the site of numerous studies of the relationship of coal smoke and lung cancer because of the unusually high rates of lung cancer in nonsmoking women using smoky coals in open fire pits (e.g., Chapman et al. 1989; Liu et al. 1991; Mumford et al. 1987). The odds ratios (ORs) for lung cancer associated with indoor coal use are summarized in a recent meta-analysis by Smith et al. (2004). For comparison, ORs were estimated with and without adjusting two important confounding factors. Tobacco smoking status was either adjusted or analyses were done solely in nonsmokers. Because chronic respiratory diseases such as chronic bronchitis, tuberculosis, asthma, and emphysema may increase the probability of developing lung cancer later in life (Luo et al. 1996), adjustment was also made for these diseases. These adjustments may result in underestimating the ORs of lung cancer, as some previous lung diseases may be on the intermediate path from exposure to lung cancer—in this case, they are not confounders and should not be adjusted. The OR estimate for women was 1.17 with 95% confidence interval (CI) of 1.02–1.35. However, when the analysis was restricted to studies that adjusted for smoking and chronic respiratory disease, the OR estimate for women substantially increased to 1.94 (95% CI, 1.09–3.47). The OR estimate for men was 1.79 (95% CI, 1.18–2.72) and slightly lower when including confounding by smoking and chronic airway disease with OR = 1.5 (95% CI, 0.97–2.46). The overall OR estimates for men and women combined were 1.86 (95% CI, 1.48–2.35) and 2.55 (95% CI, 1.58–4.10), respectively, both without and with the two confounding factors adjusted. In a more recent review, Zhao et al. (2006) found similar results.

The meta-analyses present strong epidemiologic evidence that exposure to indoor coal smoke significantly increases lung cancer risk. Recently, the International Agency for Research on Cancer (IARC) classified emissions of indoor combustion of coal as carcinogenic to humans (Group 1) on the basis of sufficient evidence both in humans and in animals (Straif et al. 2006). The role of certain genotypes and proteins in the development of lung cancer has been examined in Xuanwei County residents using smoky coal, suggesting that an individual’s susceptibility to lung cancer may be increased by the glutathione *S*-transferase 1 (*GST1*)-null genotype (Lan et al. 2000, 2001; Lan and He 2004; Mumford et al. 1999). There is also limited evidence that other cancers can be caused by exposure to household coal smoke, including esophageal (Pan et al. 1999) and cancers of the head and neck (Dietz et al. 1995). This may be due to not only direct respiration of airborne pollutants but also the contamination of food by coal smoke (Roth et al. 1998).

### Respiratory illnesses

Indoor coal smoke exposure has been linked to various respiratory illnesses. A study conducted in Anhui Province shows that the prevalence rates of chest illness, cough, phlegm, and shortness of breath were significantly elevated in nonsmoking women living in homes with both smokers and coal heating (Pope and Xu 1993). A survey of 10,892 Xuanwei residents found that the OR estimates for smoky coal users compared with smokeless coal users, were 1.73 for shortness of breath, 3.30 for cough, and 4.23 for phlegm, and that the OR estimates for smokeless coal users compared with wood users were 1.35 for cough and 1.67 for phlegm (Zhou et al. 1995). A study of 5,051 seventh-grade students from 22 randomly selected schools in the greater metropolitan area of Wuhan found that coal burning for cooking/heating increased the risk of wheezing with colds (OR = 1.57; 95% CI, 1.07–2.29) and without colds (OR = 1.44; 95% CI, 1.05–1.97) (Salo et al. 2004). In a population-based case–control study of childhood asthma conducted in Shunyi County located in suburban Beijing, an increased risk was observed for use of coal for heating (OR =1.5; 95% CI, 1.1–1.9) and for use of coal for cooking without ventilation (OR = 2.3, 95% CI, 1.5–3.5) (Zheng et al. 2002). Indoor coal use was associated with increased incidence of rhinitis, faucitis, and tonsillitis in children living in Taiyan City, Shanxi Province (Cheng et al. 2002). The effects of household coal use were also observed in 624 infants and young children (1–3 years of age) in Nantong, Jiangsu Province, as the prevalence of cough and that of pneumonia were significantly higher in households using coal than in the “control” households using gas (Zhou et al. 1994). Chronic obstructive pulmonary diseases (COPD) is a major cause of ill heath in China, causing more than 1.3 million deaths annually (WHO 2002). A case–control study conducted in Shanghai showed that indoor coal use was more strongly associated with COPD than estimated exposure to outdoor SO_2_ and PM_10_ (Tao et al. 1992). A survey of 21,648 rural residents in Anhui Province showed that the COPD rate was significantly higher in individuals who used coal for heating than in those who did not (Li et al. 2002).

Exposure–response relationships have been examined in a study of 7,058 elementary school children living in four large Chinese cities. When lifetime exposures to coal smoke from heating were classified according to four ordinal levels (no, light, moderate, and heavy exposure), monotonic and positive exposure–response relationships were observed for OR estimates of phlegm, cough with phlegm, and bronchitis. In addition, OR estimates for cough, wheeze, and asthma were all > 1 in the exposed groups relative to the no-exposure group (Qian et al. 2004).

### Lung function reduction

The effect of coal smoke exposure on lung function has been investigated in a few studies of children or adults. In school children living in the cities of Chengde (Hebei Province) and Shanghai, measurements made in winter showed reductions of 1.5–10.7% in forced vital capacity (FVC), forced expiratory volume in 1 sec (FEV_1_), or peak expiratory flow rate (PEFR). These reductions were associated with the use of coal for cooking/heating compared with the use of natural gas or LPG (Shen et al. 1992). In adults, evidence of lung function impairment from coal smoke has been found in a few studies (Jin et al. 1995; Wang H et al. 2004; Xu et al. 1991). In 1986 when household coal use was still prevalent in Beijing, lung functions of 1,440 of the city’s adult never-smoking residents were measured (Xu et al. 1991). The authors found that heating with coal stoves was associated with reduced FEV_1_ and FVC compared with radiator-based heating supplied from a centralized boiler. Simple and inexpensive PEFR measurement can be self-conducted by subjects, making a study possible to measure a large number of people, such as the one conducted in 10,892 adults living in 18 villages of Xuanwei County. This study compared the relative potencies of smoky coal, smokeless coal, and wood and examined the effect of these on PEFR. The results show that the strongest risk factor for lowered PEFR was smoky coal, followed by smokeless coal, then by wood. The study also found that the use of coal stoves with chimneys was associated with increased PEFR compared with the use of open fire pits (Jin et al. 1995). In a recent study conducted in Shanxi Province, women using coal for cooking had lower lung function values than those using gas for cooking (Wang H et al. 2004).

### Immune system impairment

Direct exposure to coal smoke or coal smoke condensate has been examined for potential effects on the human immune system in about half-dozen studies, all published in Chinese journals (e.g., Jin et al. 2002). In nonsmoking Shanghai women, those who used coal for cooking had significantly lower serum IgG content, peripheral T-lymphocyte activity, E-rosette formation rate, and interleukin (IL)-2 induction activity than those who used gas for cooking (Wang et al. 1993). Similar findings were reported in another article that additionally reported decreased activity of natural killer cells in women using coal for cooking, although no significant association was found for IL-2 induction activity in T-lymphocyte cells (Mao et al. 1994). A study of 624 infants and young children found that serum IgG content was significantly lower in those whose households used coal for cooking than in those whose households used gas fuels (Zhou et al. 1994). These findings suggest that coal smoke exposure weakens the human immune system, making those exposed individuals more susceptible for developing illnesses (Jin et al. 2002).

### Poisonous coal endemics

In China there are approximately 100 counties (of approximately 1,500) that have been deemed “endemic” because local coal deposits have high contents of toxic elements (Sinton et al. 2004). The most noticeable coal-related endemics are arsenism and fluorosis, which are the result of chronic arsenic and fluoride poisoning. Burning arsenic-rich coals occurs widely at least in eight counties of two provinces (Guizhou and Shaanxi), affecting approximately 300,000 people (He et al. 2005; Jin et al. 2003). Reported illness includes symptoms of arsenicosis (Shraim et al. 2003).

It is known that high arsenic exposure via drinking water causes bladder, lung, and skin cancers (Boffetta 2004). However, cancer has not been studied in poisonous coal areas in China. It is estimated that more than 10 million people in Guizhou Province and surrounding areas suffer from dental and skeletal fluorosis (Cao 1991; Chen et al. 1993; Finkelman et al. 1999), mainly as a result of excess fluoride intake from eating foods dried over open fire pits (Wu and Li 1990; Yan 1990). In some fluorosis areas, almost all elementary and junior high school students had dental fluorosis, and osteosclerosis in the skeletal fluorosis patients was very serious (Ando et al. 1998, 2001; Watanabe et al. 1997). In addition, chronic selenium and possibly mercury poisoning has also been reported to result from household coal use in the affected areas (Fang et al. 2004; Finkelman et al. 1999; Horvat et al. 2003).

### CO poisoning

There have been numerous reported acute poisonings, including fatal cases, especially during heating seasons, resulting from indoor coal combustion under poor ventilation conditions. Under normal combustion and ventilation conditions, Zhang X et al. (1996) measured elevated blood levels of CO–hemoglobin adduct (COHb) in residents of households using coal and reported that the contribution to COHb from indoor coal combustion was larger than that from cigarette smoking. However, the health effects of chronic CO exposure at elevated levels but lower than those of acute poisoning have not been examined in China, although this type of CO exposure is typical in households using solid fuels (Zhang et al. 1999). CO is a known neurotoxin, and there is a potential for chronic exposure to exert neurologic effects. Furthermore, it has been associated with effects on prenatal and early postnatal mortality and low growth in children of women exposed during pregnancy. These effects are presumably due to oxygen deprivation.

### Evidence from intervention studies

Assignment of causality is difficult on the basis of observational studies alone, the interpretation of which is limited by potential unmeasured confounders, for example, socioeconomic status, which is often associated with disease outcome as well as use of dirty household fuels. No randomized intervention trials providing stronger evidence have yet to be published for household solid fuel use in China and would be difficult to conduct for chronic diseases such as COPD and lung cancer that are the results of many years of exposure. Using the rich database of studies in Xuanwei, however, two intervention studies (Chapman et al. 2005; Lan et al. 2002) have been published that take advantage of the “natural experiment” when improved coal stoves with chimneys were introduced in the late 1970s to replace open fire pits, thereby lowering indoor air concentrations by a factor of approximately 3 on average (Lan et al. 2002). A questionnaire was administered in 1992 to more than 10,000 farmers in the area upon which the following two studies were based.

#### Lung cancer

In a retrospective study of 21,232 farmers between 1972 and 1992, during which 17,184 shifted to improved stoves, hospital records indicated 1,384 cases of lung cancer (Lan et al. 2002). Cox-modeled risk ratios (RRs) for lung cancer resulting from stove intervention were 0.59 (95% CI, 0.49–0.71) for men and 0.54 (95% CI, 0.44–0.65) for women. Spending more time indoors, cooking history, and living in a larger family were also associated with lung cancer. Living in a house with more than three rooms was protective.

#### COPD

The 1976–1992 COPD histories of 20,453 farmers were determined by asking whether they had ever been diagnosed with chronic bronchitis or emphysema and by death records (Chapman et al. 2005). Of these, 16,606 had installed improved stoves and 1,487 had COPD. Cox-modeled RRs of the stove improvement were found to be 0.58 (95% CI, 0.49–0.70) in men and 0.75 (95% CI, 0.62–0.92) in women.

It is noteworthy that the RRs for both lung cancer and COPD incidence decreased over time since the stove improvement; and that the risk reduction became nearly complete after about 10 years for both. These studies differ from others, however, in that the benefits of stove improvement for men were similar (for lung cancer) or greater (for COPD) than for women who are usually thought to have higher exposures from stove emissions. For the lower COPD effect, we offer the explanation that cooking exposures start early in life, and thus women may have been compromised before the intervention. We also point out that even after intervention, indoor levels of PM_10_, approximately 700 μg/m^3^, were still high (Chapman et al. 2005). If one assumes, however, that the relative decrease in exposure was similar for both sexes but that women experience a generally higher exposure level because of their role as cooks, then a lower effect in women might be interpreted as a shallowing of the exposure–response curve. These are pioneering studies in the IAP field, the first of their kind in the world, and move the level of evidence of harm to new levels of sophistication and credibility. There are few such studies even among the thousands of outdoor epidemiologic studies of air pollution health effects.

## Interventions

The infamous smog episode killing thousands of people in the winter of 1952 eventually led to the ban of household use of coal fireplaces in London. Today, in the United Kingdom and other developed countries, household use of coal in cities is almost nonexistent. With the rapid economic growth in China, coal stoves are becoming less common in cities, as they are being replaced with gas stoves and with space heating methods other than direct coal combustion. Despite the declining trend, however, household coal use is still common in urban communities across China. In rural China, coal use seems to be increasing as coal substitutes for biomass, and there have been proposals by national and international agencies to promote household coal use, usually in the form of “clean coal,” to reduce the growing use of petroleum-based fuels and to relieve the pressure on biomass resources. Although household use of piped gas is increasing in cities and LPG use is increasing among affluent rural households, widespread use of gas fuels in rural households is unlikely to occur soon because of the cost and the unreliability of supplies. Hence, interventions to make solid fuels less polluting continue to be important for public health.

### Fuel/stove interventions

Because pollutant emissions depend on both fuel quality and stove design, interventions can focus on fuel, stove, or both. To date, stove interventions have focused predominantly on households using biomass, and fuel interventions appear to have focused mainly on coal, although the reverse trends are also becoming apparent.

The most impressive organized rural energy intervention in human history was China’s NISP, through which China accomplished more than all other developing countries combined to improve household energy use by introducing more than 180 million improved stoves since the early 1980s. All introduced stoves had chimneys and some had manual or electric blowers to promote more efficient combustion. Unfortunately, the program ended in the mid-1990s, and now there is relatively little action to improve the present rural energy situation. The review of NISP by Sinton et al. (2004) found that the program improved IAQ, but not sufficiently to meet China’s IAQ standards. In addition, because the NISP focused mainly on biomass, rising coal use in rural areas, often in stoves without chimneys, is threatening to erode the benefits. Although the Chinese Ministry of Health has embarked on a program to introduce improved coal stoves to approximately 100 endemic arsenism and fluorosis counties, progress has been slow because of the lack of resources (Sinton et al. 2004).

Various formulated coals have been developed in China to reduce hazardous emissions. The most noticeable is the formulation of so-called honeycomb coals that have been used widely throughout the country for decades. The perforated shape of the coal allows for more uniform air supply, consequently leading to higher combustion efficiency. Some honeycomb coals are specially formulated, for example, with lime to react with fuel sulfur and retain it in ash, instead of emitting it as SO_2_ (Ge et al. 2004). Because of different coals, different formulations, and different measurement methods, it is difficult to generalize about the impact of such coal fuels (Bond et al. 2002; Kasper et al. 1999; Perlack and Russell 1991; Yao et al. 1992).

In some areas, “clean” forms of coal are being promulgated for urban use, but their sustainability is uncertain. In addition, household coal fuels are not required to undergo standard testing, and there is even less regulation of what is actually sold in the market place. In rural China little attention is focused on clean coal, and household coal varies dramatically across the country according to the character of local coal deposits. In developing clean coal strategies, it is useful to consider some historical lessons from other parts of the world. Before actually banning coal in cities, the United Kingdom and other countries using household coal developed and deployed a range of clean coals for small-scale use. This might have helped lessen the pollution problem in the short-term but, eventually, these countries realized that in simple household combustion, even the processed forms of coal or cleaner natural forms, such as anthracite, could not be burned clean enough to use in urban areas and still meet health-based pollution standards. Perhaps the question now is how clean is today’s clean coal compared with those clean coals used half a century ago.

### Emerging technologies

In rural China there is the potential for modernization of its rich biomass resources into clean energy sources. The generation rate for crop residues in the field plus agricultural processing residues amounts to about 790 million metric tons per year, or approximately 10 exajoule (EJ). For comparison, total coal use in China was 39 EJ in 2004 in all sectors (National Bureau of Statistics 2006). It has been estimated that about half the total crop residues might be available for energy use after accounting for other uses (e.g., fodder, fertilizer, and industrial feedstock). An alternative to traditional direct combustion of crop residues is the use of “gasifier” stoves that achieve high combustion efficiency through designs that promote secondary combustion. Reliably high combustion efficiency is easier to accomplish with small electric blowers, but some models attempt to do so with natural draft. A national competition was held in 2006 by the Chinese Association of Rural Energy Industries funded by the UK Shell Foundation to promote the development and dissemination of such low-emission biomass stoves and fuel cycles. To reliably achieve low emissions in field conditions, however, such stoves require more uniform fuel through, perhaps, development of small local biomass-processing enterprises. Stoves burning biomass pellets, for example, can achieve remarkable efficiencies.

Recently, new pollution problems are arising from crop residues because in more economically developed parts of the country, farmers are becoming reluctant to gather biomass residues from the field and store them for use throughout the year, preferring easier-to-handle fuels such as coal briquettes or LPG. This shift to modern fuels creates an excess of crop residues that are commonly burned in the field, leading to widespread ambient pollution in some seasons. A number of village-scale gasifiers have been built to make more efficient and cleaner use of these residues by distributing the gas to individual households. A Bio-energy Modernization Demonstration Project, carried out in Jilin Province, is designed to develop combined heat, electricity, and cooking fuel production (trigeneration) from corn stalks. The technology seems to be workable, but its widespread use, however, is limited by the lack of commercial viability and potential risks due to acute CO poisoning (United Nations Development Program et al. 2004).

Simple household biodigesters have long been available for converting animal waste into biogas containing methane, but these biogesters are limited to areas with sufficient dung, water, temperature, and financial capital. Biobriquette technology, in which biobriquettes are made of biomass, coal, and sulfur fixation agents through high-pressure manufacturing processes (Lu et al. 2000), is being investigated. Such a fuel has shown an acid-neutralizing capacity of the ash it produces (Dong et al. 2004) and a reduction in SO_2_ emission (Isobe et al. 2005).

Advanced technologies are also being explored for converting crop residues into dimethyl ether (DME), a fuel with characteristics similar to those of LPG. DME, sold as a substitute for LPG, is currently made from natural gas but can only take on a large role if made from crop residues or coal. Finding ways to convert coal, the country’s most abundant energy resource, to DME has been attractive to researchers and the Chinese government (Han et al. 2004). With further cost reduction in coal-to-DME technologies and the rise in LPG costs, however, coal-derived DME for household use may become economic (Larson and Yang 2004). Produced with polluting and inefficient methods, the use of traditional coal gas in Chinese cities has been declining.

### Intervention benefits

In large Chinese cities, policies of banning household coal use have been in place with the main goal of reducing outdoor air pollution but with the side benefit of reducing IAP levels. Although health benefits have not been directly reported or measured in Chinese cities, the findings from cities in other countries; for example, studies of the ban on coal sales in Dublin, Ireland, documented sudden decreases in atmospheric concentrations of PM and SO_2_ as well as significant reductions in cardiovascular and respiratory death rates (Clancy et al. 2002).

Simple stove improvement is not expected to reduce total pollutant emissions because chimneys divert only a fraction of the emitted pollutants from the inside of households to the outside, rather, it worsens neighborhood and local air quality. A more permanent solution is to move to clean combustion through changes in fuel and/or stoves, which actually reduce emissions. An added benefit from clean combustion is improved atmospheric visibility and lower ambient pollution as well as potentially lower greenhouse emissions. PICs from low-efficiency biomass combustion in household stoves, for example, contain a relatively large fraction of methane many times more potent in radiative forcing than CO_2_, implying that biomass stoves can have net global warming potentials, even with a renewable fuel cycle (Smith et al. 2000).

## Conclusions and Recommendations

Solid fuels are still the dominant source of energy in Chinese households, leading to pollutant levels generally exceeding China’s IAQ standards and contributing significantly to the national burden of ill health. Evidence for adverse health outcomes is strong, including lung cancer, respiratory illnesses, acute respiratory infection, and COPD. There is also evidence in China of impacts on lung function and immune system impairment. Therefore, improving IAQ in households using solid fuel should be an urgent and high-priority task on China’s public health agenda. A range of intervention technologies, from one as simple as adding a chimney to the more complex modernized bioenergy program, is available, but these technologies can be viable only with a coordinated support from the government and interested private parties in the commercial sector. Substituting cleaner fuels for the poisonous coals being used in millions of households should have an especially high priority. This was one of the principal recommendations from a Chinese and international scientific and policy workshop convened in early 2005 to review the status of improved stoves in China. The full set of recommendations and related materials can be found in Smith (2005).

## Figures and Tables

**Figure 1:**
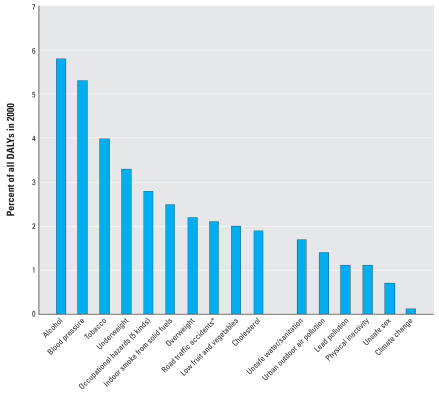
Rough estimates of the burden of disease in China: the top 10 risk factors plus other selected risk factors [adapted from Smith et al. (2005)]. Note: Indoor smoke from solid fuels does not include smoke from other fuels or tobacco. Burden of disease is measured as disability-adjusted life-years (DALY) including those lost to premature death and those lost to illness as weighted by a disability factor (Ezzati et al. 2004). Such estimates are associated with relatively large uncertainties because the data available on pollution exposure and on exposure–effects relationships are rather limited for China, despite the apparently large risks and populations involved. *Data from Smith and Ezzati (2005).

**Table 1:** China’s indoor air quality standards.*a*

Pollutant	Maximum allowable level	Averaging time
PM_10_	150 μg/m^3^	1 day (24 hr)
SO_2_	500 μg/m^3^	1 hr
NO_2_	240 μg/m^3^	1 hr
CO	10 mg/m^3^	1 hr
Formaldehyde	100 μg/m^3^	1 hr
B[*a*]P	1.0 ng/m^3^	1 day (24 hr)

a Data from SEPA (2007). Only the pollutants most relevant to solid fuel combustion are listed.
